# Stupidity

**Published:** 1859-05

**Authors:** 


### STUPIDITY.

THE stupidity of people as to some of the commonest things of life is amazing, except to the few who are wise enough to have no amazement, having taken it for granted that not one man in a thousand has sense enough to see an inch beyond his own nose out of his immediate business.

As there are more than twenty thousand post offices in the United States, it would seem that any man at a small remove from an idiot, would know that if he wrote a letter requiring an answer, it was of some consequence to state to what town and county and state it should be sent. But we are constantly receiving letters, with various amounts of money, either for advice or books, or information, without any direction explicit enough to warrant any reply.

It is well known that youths from fifteen to twenty, and fools of all ages, are the smartest people in the world; they know everything; they never perform an act which is not perfectly just—in their own estimation, and no Nero is so impatient and so savagely severe in punishment if anything is done contrary to their views of what ought to be done.

Not long ago, a lawyer sent us some money, and we kept it, because there was no place named at the head of his letter to which we could send what he wanted. In due time another letter came in the same way, with the same amount of money, and we kept it. He stated that he had been practising law forty years in the town, and was so well known that if a reply had been sent he would have received it, but being of age he did not get mad, but concluded the first letter had miscarried.

Later, a clergyman writes: the name of his town is plainly written, but no state, and there are seven such towns in the Union; and not choosing to write seven letters, with the loss of their postage, paper and envelopes, in endeavoring to find out where to send a ten cent journal, we concluded to wait for another letter, and here it is,—no county or state named in the letter, or on the envelope, although it is the sworn duty of the post-master to place the name of the post office address, as to town and state on each letter or envelope.

"If you are disposed to treat this note with silent contempt, as you have its predecessor, then we will say you are entirely welcome to the Yankee shilling. Let the matter rest here and hereafter. So mote it be."

Let such a letter, from such a source, teach our patrons this little lesson,—never blame anybody for not answering a letter until you are perfectly satisfied and know—

First, That you have given explicit directions as to what place the answer is to be sent, or

Second, That you sent an envelope, and your full address written thereon, with a stamp for return postage.

Third, That your letter was not impertinent or unreasonable or discourteous, and even then have the charity to remember that your letters may have miscarried, or that the replies to them may have miscarried, or that the person to whom you wrote may have been from home, or sick, or was so engaged that the reply could not be attended to without such a neglect of his own business as you would not yourself wish to have occasioned.

In short, reader, never make a fool of yourself by writing a letter while in a passion, to any body, however high or however low. And even if under great provocation, take a noble pride in exhibiting the dignified courtesy of a gentleman, and the forbearance of a christian; and remember, the more you gloat over the severity of what you have written, the more of an ass you will be for sending or publishing it.

